# Optical coherence tomography: evaluating the effects of stent boost subtract imaging on stent underexpansion in STEMI patients

**DOI:** 10.1186/s12872-022-02498-9

**Published:** 2022-02-20

**Authors:** Yuanyuan Duan, Limin Jing, Shi Pan, Sujuan Yan, Fang Wang, Hong Yu, Beibei Zhang, Guangsheng Wei, Ming Zhang, Jiao Zhang

**Affiliations:** 1 The Second Medical Center&National Clinical Research Center of Geriatric Diseases, Chinese PLA General Hospital, Beijing, 100039 China; 2 Department of Cardiology, Beijing Electric Power Hospital, State Grid Corporation, #Jia 1 Taipingqiaoxili, Fentai, Beijing, 100073 China

**Keywords:** Optical coherence tomography, Stent boost subtract imaging, Acute myocardial infarction, Stent underexpansion

## Abstract

**Background:**

To evaluate the effect of stent boost subtract (SBS) imaging on stent underexpansion during percutaneous coronary intervention (PCI) in patients with acute ST-segment elevation myocardial infarction (STEMI) by optical coherence tomography (OCT).

**Methods:**

One hundred thirty-eight STEMI patients who underwent drug-eluting stent (DES) implantation were prospectively recruited and divided into the SBS group (69 cases) and the CAG group (69 cases) according to whether SBS was used to guide PCI. Finally, OCT was performed on all enrolled patients, and the OCT results were used as the gold standard to evaluate the impact of standard SBS technology on stent underexpansion immediately after DES implantation.

**Results:**

SBS identified 51 patients (24%) with stent underexpansion while OCT identified 56 patients (27.2%). SBS has a sensitivity of 80%, a specificity of 96%, a positive predictive value of 88%, and a negative predictive value of 93% for identifying stent underexpansion.

**Conclusion:**

Compared with OCT, SBS technology is a rapid stent imaging evaluation method that can accurately quantify the stent expansion level and is time-saving and economical.

**Supplementary Information:**

The online version contains supplementary material available at 10.1186/s12872-022-02498-9.

## Introduction

Percutaneous coronary intervention (PCI) is currently the most important method for the treatment of acute ST-segment elevation myocardial infarction (STEMI) [1, 2]. However, the occurrence of stent underexpansion and stent malapposition has significantly increased the incidence of in-stent thrombosis after PCI [3, 4]. Therefore, there is an urgent need for a method that can accurately determine stent expansion to guide stent postexpansion to optimize PCI strategies and improve PCI prognosis in STEMI patients. Intravascular ultrasound (IVUS) is considered to be the gold standard to evaluate stent expansion due to its ability to accurately quantify the level of stent expansion [5]. Compared with IVUS, optical coherence tomography (OCT) is an intracavity imaging technique with higher resolution. Therefore, using OCT to determine stent expansion has a significant advantage in terms of resolution/image quality over IVUS [6, 7]. Although IVUS and OCT can identify stent underexpansion that cannot be detected by coronary angiography (CAG), they have not been widely used to guide PCI in STEMI patients due to the complexity of the operation, the high cost, and the need for experienced interventional personnel to perform the operation [8, 9]. Stent boost subtracts (SBS) is a newly developed imaging technology. Based on coronary angiography, the continuous frame images are superimposed and converted into a digital movie, and finally, the enhanced image of the stent and the blood vessel wall is obtained. As a result, stent underexpansion or stent malapposition can be detected in time [10]. The purpose of this study was to use OCT as the gold standard to evaluate the effect of SBS on stent underexpansion in guiding PCI in patients with STEMI.

## Materials and methods

### Study population

**Fig. 1:**
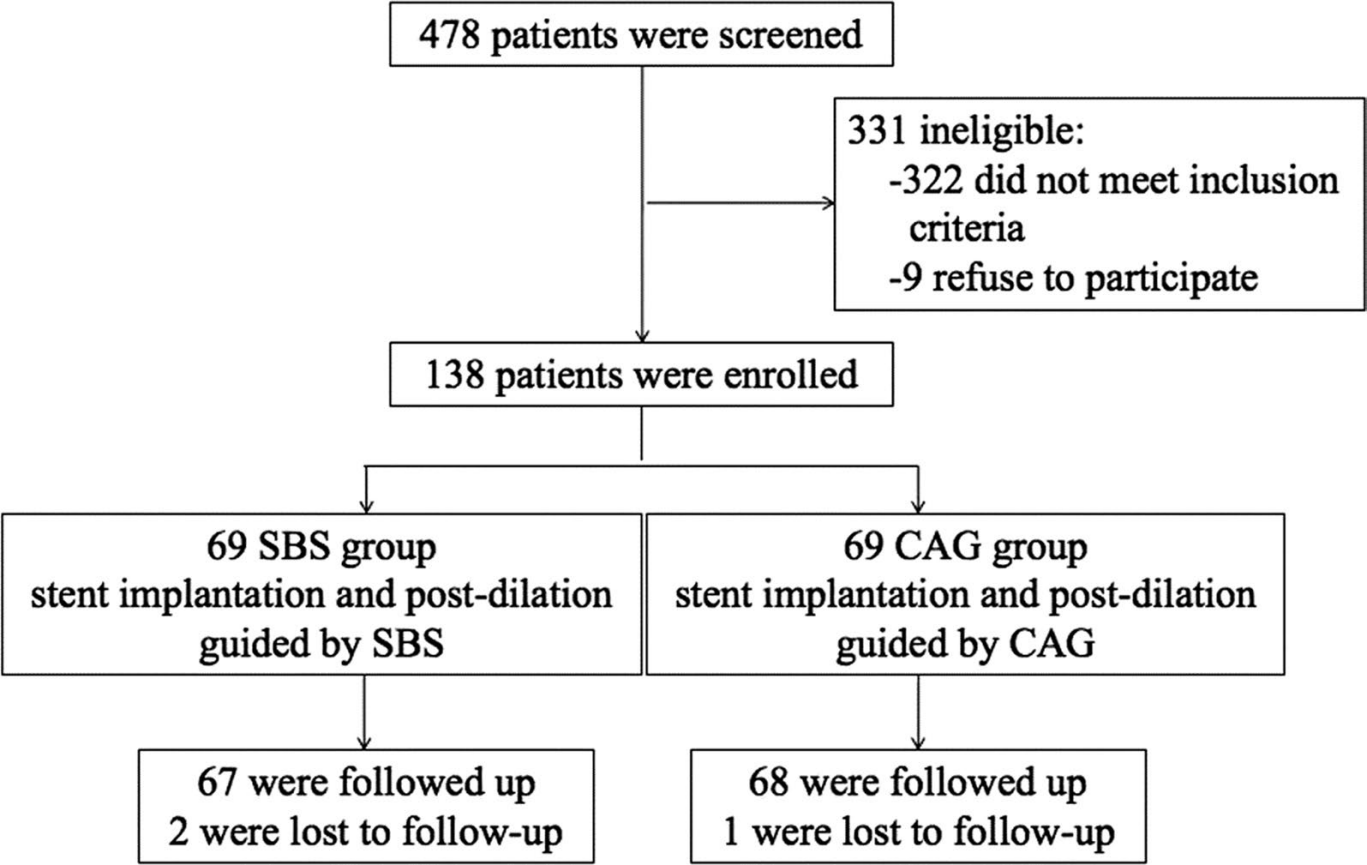
Participants selection and groups. *CAG* coronary angiography, *SBS* stent boost subtract

This trial was a prospective, observational study. The trial profile is shown in Fig. 1. From June 2015 to March 2019, STEMI patients who received EXCEL™ rapamycin drug-eluting stent (DES) implantation were recruited from the Department of Cardiology, Armed Police General Hospital. The participants were divided into the SBS group (69 cases) and CAG group (69 cases) according to whether the SBS was used to guide PCI (Fig. 1). Patients were eligible if they had ≤ 2 lesions with 75% diameter stenosis in a native coronary artery, with a reference vessel diameter between 2.5 and 4.0 mm. Exclusion criteria: (1) Left main coronary artery lesion, (2) Target lesions within 10 mm from the side-branch opening, (3) Visually inspect the target lesions with side branch vessel diameters > 2.0 mm or < 2.0 mm that require treatment, (4) Chronic total occlusion, (5) Failure to complete OCT examination, (6) severe complications that can result in death and myocardial injury or require immediately invasive treatment, (7) NYHA grade ≥ Grade III, and (8) renal insufficiency (creatinine > 1.5 mg/dL).

### Operation procedure

Before PCI, the baseline information, symptom characteristics, and the number of lesions of each patient were evaluated. Then, DES was implanted into the target lesion. In the SBS group, SBS was used to evaluate the stent expansion immediately after the DES was implanted, and according to the SBS results, postdilation was performed or not until SBS indicated that the stent expansion was optimal. In the CAG group, coronary angiography was used to assess the expansion of the stent, and the process was the same as that in the SBS group. Both groups underwent OCT examination immediately after PCI and after postdilation. After the angiography, the interventional physician of our Intervention Center will complete the stent implantation. During the operation, the stent will be preprocessed according to the disease condition and the experience of the surgeon, and then the stent will be implanted to cover the lesion. After stent implantation, the surgeon will be based on the expansion of the stent and decide whether to perform poststent expansion. All patients in the SBS group used SBS technology to guide stent positioning and poststent expansion. Finally, the data were collected and analyzed offline by two independent investigators.

### SBS protocol

The Allura Xper FD20 digital flat panel angiography system (Philips Medical Systems, Netherlands) was used in digital angiography and fluoroscopy. The operation process was as follow: first, put the balloon with two markers in the stent, without injecting the contrast media, step on the exposure for 2–3 s, and then inject the contrast media to expose for 2–3 s. Second, SBS automatically converts the continuous frame superposition collected by dynamic compensation into a digital movie, so that the stent can be autoradiographed. Finally, the image was automatically transferred to the workstation for offline SBS image analysis. Analysis indicators included the minimum stent diameter, the maximum stent diameter, and the mean and variance of the inner diameter of the stent. The criteria of an optimal stent expansion included (1) good attachment of the stent: the edge of the stent column was parallel to the vessel wall, and there was no gap between the stent and wall; (2) no sign of local underexpansion of the stent; and (3) the minimum stent diameter ≥ 2.5 mm.

### OCT assessment

OCT system (C7 system; St. Jude Medical, St. Paul, USA) was immediately used to evaluate stent expansion after SBS. After auto-calibration, the OCT imaging catheter was placed in the lesion and was automatically withdrawn to obtain the image. Then, the OCT image data were offline analyzed. The stent expansion rate was equal to the minimum stent area/mean reference lumen area × 100%. The stent underexpansion criterion was a stent expansion rate < 90%. The stent symmetry index was equal to (maximum stent diameter minus minimum stent diameter) divided by the maximum stent diameter.

### Follow-up after PCI

At 1, 3, and 6 months after PCI, the patients were followed up in outpatient clinics or by telephone to record the occurrence of major adverse cardiovascular events (MACE) which were defined as acute myocardial infarction, target vessel revascularization and angina pectoris. The patients were followed up with angiography 9 months after PCI. The minimum stent diameter, the minimum lumen diameter of the proximal and distal segments of the stent, the late lumen loss and restenosis in the stent and intrasegment were recorded.

### Statistical methods

The sample size was calculated by PASS sample size calculation software. There has been no previous research on the effect of SBS-guided DES implantation evaluated by OCT. According to a previous study, the incidence of stent underexpansion immediately after PCI evaluated by OCT was 65%. We estimated that with a total of 114 patients (57 cases in each group), the study would have 80% power to show noninferiority, with a two-sided type I error rate of 0.05. In addition, considering the maximum loss rate of 20%, 138 patients were ultimately required to be able to distinguish the difference between the two at least. All data were analyzed statistically using SPSS 20.0. Count data are expressed as frequency and percentage. Baseline data meeting a normal distribution are expressed as the mean and standard deviation, and a t-test of two independent samples or paired samples is adopted to test the differences. Data of nonnormally distributed variables are expressed as medians and interquartile ranges, and a nonparametric test was adopted to test the differences. Kappa test was used for consistency test. Since the same patient may have multiple target vessel diseases, the generalized linear mixed-effects model was used to compare the differences between groups, with the measured value as the outcome variable and the patient and the lesion as random intercepts, with grouping as fixed effects. *P* < 0.05 is considered statistically significant.

## Results

### Baseline characteristics of patients

A total of 138 patients with coronary heart disease who underwent DES implantation in our hospital from June 2014 to March 2018 were recruited. Two hundred and fourteen lesions were included in the image analysis. The clinical baseline characteristics of the patients are shown in Table 1. The target lesion characteristics and stent-related information are shown in Table 2.

**Table 1:** Clinical baseline characteristics of the patients

Demographic characteristics	CAG group (N = 69)	SBS group (N = 69)	*P* value
Age (years)	61.7 ± 8.6	60.3 ± 9.8	0.374
Sex (male) (%)	41 (59.4)	46 (66.7)	0.380
Hypertension (%)	45 (65.2)	49 (71.0)	0.465
Diabetes mellitus (%)	21 (30.4)	25 (36.2)	0.470
Hyperlipidemia (%)	58 (83.1)	54 (78.3)	0.758
Family history of CHD (%)	36 (52.2)	30 (43.5)	0.307
Current smoking (%)	42 (60.9)	37 (53.6)	0.390

*CHD* coronary heart disease

**Table 2:** Angiographic data of the patients

Angiographic characteristics	CAG group (N = 69)	SBS group (N = 69)	*P* value
Diseased vessel			
LAD	33 (47.8)	34 (49.3)	0.865
CX	25 (36.2)	22 (31.9)	0.590
RCA	23 (33.3)	26 (37.7)	0.594
Type B2/C lesions	27 (39.1)	34 (49.3)	0.230
Mean number of stents	1.32 ± 0.47	1.35 ± 0.72	0.772
Stent diameter (mm)	3.12 ± 0.38	3.15 ± 0.67	0.747
Mean stent length (mm)	24.31 ± 7.2	24.92 ± 5.3	0.572
Overlap stenting	27 (39.1)	18 (26.1)	0.102
Post dilation	44 (63.8)	51 (73.9)	0.198
Preoperative QCA analysis			
Reference diameter (mm)	2.86 ± 0.34	2.86 ± 0.48	1.000
Minimal lumen diameter (mm)	0.97 ± 0.28	0.91 ± 0.33	0.252
Diameter stenosis (%)	71.39 ± 8.59	72.47 ± 9.73	0.491
Lesion length (mm)	24.17 ± 8.78	24.26 ± 8.19	0.950
Postoperative QCA analysis			
Minimal stent diameter (mm)	2.91 ± 0.43	2.98 ± 0.31	0.275

*LAD* left anterior descending artery, *CX* circumflex coronary, *RCA* right coronary artery

### Stent underexpansion assessment

A total of 206 lesions were included in SBS and OCT image analysis. At the frame level, after excluding 158 frames of poor-quality images, a total of 5147 frames of images were included in the analysis. SBS identified 51 cases (24.8%) of stent underexpansion, while OCT identified 56 cases (27.2%) of stent underexpansion, with a kappa value of 0.789 (*P* < 0.001). SBS has a sensitivity of 80%, specificity of 96%, positive predictive value of 88%, and negative predictive value of 93% for identifying stent underexpansion (Additional file 1: Table S1).

### 9-months follow-up

Among the 138 patients, 2 patients had slow coronary flow during PCI, and both were in the CAG group. During the follow-up period of 9 months, 135 patients (97.8%) were followed up by telephone or outpatient clinic, and 87 patients (63.0%) were followed up with angiography. Nine patients in the CAG group and 2 patients in the SBS guidance group suffered major adverse cardiac events. In the CAG group, 2 patients had an acute myocardial infarction, 4 patients had target vessel revascularization at the 9-month follow-up, and 3 patients were readmitted to the hospital due to angina pectoris. In the SBS guidance group, 1 patient underwent target vessel revascularization at the 9-month follow-up, and 1 patient was readmitted to the hospital due to angina pectoris. The incidence of MACEs in the SBS group was significantly lower than that in the CAG group, and the difference was statistically significant (*P* < 0.05), as shown in Table 3.

**Table 3:** Cumulative incidence of adverse events among the two groups of patients during the nine-month follow-up

Adverse events	CAG group (N = 67)	SBS group (N = 68)	χ^2^ value	*P* value
MACE n (%)	9 (13.4)	2 (2.94)	4.964	0.026
AMI	2 (2.98)	0		
TVR	4 (5.97)	1 (1.47)		
AP	3 (4.47)	1 (1.47)		

*MACE* major adverse cardiac events, *AMI* acute myocardial infarction, *TVR* target vessel revascularization, *AP* angina pectoris, *CAG* coronary angiography, *SBS* stent boost subtract

## Discussion

A previous study has showed that more than half of in-stent thrombosis was caused by stent underexpansion [3], and reasonable postexpansion can effectively reduce the incidence of stent underexpansion [11, 12]. Stent boost (SB) improved angiographic visualization of the stent and of its relationship with the corresponding vessel lumen by enhancing the X-ray focus of the local region. This provides a reasonable basis for stent postexpansion [10]. According to our results, we found that in the same group of patients, compared with OCT, SBS has a good consistency in identifying stent underexpansion. SBS has a sensitivity of 80%, a specificity of 96%, a positive predictive value of 88%, and a negative predictive value of 93% for identifying stent underexpansion. Therefore, SBS can effectively identify the occurrence of stent underexpansion after drug-eluting stent placement in STEMI patients. Compared with IVUS and OCT, SBS has several significant advantages [13, 14]: First, SBS does not require additional devices to be placed in the coronary arteries, avoiding related mechanical complications; second, the technology is almost completely automated without the need for additional training of staff in catheterization laboratories; finally, compared with traditional coronary angiography, SBS will not increase the procedural time and additional costs. Therefore, SBS technology is a rapid stent imaging processing technology that can accurately quantify the level of stent expansion while saving time and cost and is valuable for clinical application.

Our recent SBS-related research showed that SBS has not significantly increased the radiation dose of patients in clinical applications [15] and further confirmed that SBS can effectively identify stent underexpansion in coronary ostial lesions [16]. More importantly, recent study found that poor stent expansion has a more pronounced effect on the prognosis of patients [17]. Therefore, in this study, we focused on the analysis of the SBS technology on the recognition effect of stent underexpansion. In this study, we found that with the continuous progress of the current stent research, the development of the platform, and the application of postoperative non-compliant balloons, the occurrence of severe acute stent underexpansion has been greatly reduced. Although SBS technology can enhance the visibility of the stent, the continuous frame superposition collected by dynamic compensation is converted into a digital movie so that the area of the stent-enhanced visualization is limited to the area within the markers at both ends of the balloon, and outside this area the image will be blurred. When using SBS technology to evaluate overlapping stents, because the total length of the stent is often greater than the length of the postexpansion balloon, the entire stent cannot be included in the stent-enhanced imaging area in one SBS, and multiple SBS evaluations are required. In addition, stent-enhanced development technology will automatically identify and detect marking points. At present, some of the latest guide wires also have marking points. In actual operation, we found that this technology caused incorrect identification of the side guide wire points, resulting in enhanced misjudgment of the developing area. This requires operators to perform additional revision operations and offline image reprocessing, which has a certain impact on the fluency of SBS operations. Therefore, SBS technology, as a two-dimensional imaging technology, has several limitations in the quantitative analysis of stent underexpansion. In addition, in the follow-up, the medications post PCI and medication compliance of the each patients were not recorded in detail, which may be one of the reasons for the difference in MACE between the two groups. Therefore, the results of this study still need to be confirmed by further large-sample studies. Of course, the results of the study provided a new clinical thought and a simple, fast, and economical imaging tool that could improve optimized PCI strategies and the long-term prognosis of patients.

## Supplementary Information

**Additional file 1: Table S1.** Stent underexpansion identified by OCT or SBS.

## Data Availability

The datasets generated and/or analyzed during the current study are not publicly available due to privacy or ethical restrictions but are available from the corresponding author on reasonable request.
