# Supplementary figures and images for: Optical coherence tomography: evaluating the effects of stent boost subtract imaging on stent underexpansion in STEMI patients
Source: BMC Cardiovasc Disord. 2022 Feb 20;22:62. doi: 10.1186/s12872-022-02498-9 (PMC8859886; doi:10.1186/s12872-022-02498-9)

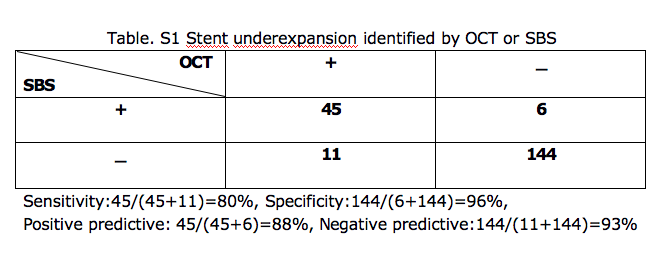

Supplement: Supplementary file 1 — Additional file 1: Table S1. Stent underexpansion identified by OCT or SBS. [file 12872_2022_2498_MOESM1_ESM.png]
